# The acronymisation of lichen sclerosus

**DOI:** 10.1002/ski2.401

**Published:** 2024-06-04

**Authors:** Georgios Kravvas, Christopher Bunker

**Affiliations:** ^1^ Department of Dermatology University College London Hospitals NHS Foundation Trust London UK; ^2^ Department of Medicine University College London London UK

During the review stage of our latest article our use of the acronym LSc was forcefully, indeed emotionally, challenged.

Lichen sclerosus is a chronic inflammatory and progressive skin disease of contested aetiology that primarily affects the genitalia.

Alternative names that have been used include lichen sclerosus et atrophicus, kraurosis, lichen albus, white spot disease, guttate scleroderma, and balanitis xerotica obliterans for the penis.

Lichen sclerosus is now the accepted terminological convention and this is endorsed by ICD codes.

An abbreviation is a truncated word; whereas an acronym is made up of parts of the phrase it stands for and is pronounced as a word (e.g., ELISA, AIDS). Abbreviations and acronyms are commonly used both in everyday clinical practice and the medical literature.

Neither ICD nor the WHO decide on medical abbreviations or acronyms, and to the best of our knowledge there is no other official body or jurisdiction for such purposes.

We accept that BAD guidelines, and some other publications, have previously used the acronym ‘LS’.^1^ But that convention does not constitute law. We, however, have preferred to use ‘LSc’ over time, and this has been accepted by journals and Editors, including recently by this journal *Skin Health and Disease* in companion publications about ‘LSc’ and melanoma.^2^, ^3^ And, indeed, ‘LSc’ is what the Editors of Rook have allowed in the new 10th edition, where lichen sclerosus is acronymised to ‘LSc’ in the male genital chapter.^4^

We contend that the use of a particular abbreviation or acronym in a scientific article should reasonably be the choice of the authors, and not be subject to irrational editorial disapprobation, and certainly not present an absolute obstacle to publication of the article. The Editors of Rook have allowed ‘LS’ in the vulval chapter, in the same volume and immediately following the male genital chapter where ‘LSc’ is used (*vide supra*).

We argue that ‘LSc’ is superior to ‘LS’ because the latter can be confused with lichen simplex, which we acronymise as ‘LSx’. Detractors have argued that ‘LSc’ might be confused with ‘LSC’, which appears from time to time in the vulval (and other) literature for lichen simplex chronicus. We argue that lichen simplex chronicus is terminologically obsolete in itself, so neither that term, nor, therefore, its acronym ‘LSC’, have a place in modern literature.

In this case we decided to concede the point to allow (a) the publication of our paper without further officious delay, (b) because ‘LS’ is how lichens sclerosus is contemporaneously acronymised in the vulval chapter in Rook and (c) to ventilate an authors’ right to choose acronyms in general within his/her work, and our argument for the use of ‘LSc’ in particular in this and our other works work, the Editor having graciously allowed us these few words.

Acronyms (and abbreviations) tend to evolve with time. But they must be comprehensible and unconfusing to be useful and safe. It would be even better if they were used consistently across the sexes in the instance of lichen sclerosus, but we rest our case.

## CONFLICT OF INTEREST STATEMENT

The authors declare no conflicts of interest.

## AUTHOR CONTRIBUTIONS

**Georgios Kravvas**: Conceptualization (equal); project administration (equal); writing – original draft (equal); writing – review & editing (equal). **Christopher Bunker**: Conceptualization (equal); project administration (equal); writing – original draft (equal); writing – review & editing (equal).

## ETHICS STATEMENT

Not applicable.

## Data Availability

Data sharing not applicable to this article as no datasets were generated or analysed during the current study.
